# The Effect of an Electronic Medical Record–Based Clinical Decision Support System on Adherence to Clinical Protocols in Inflammatory Bowel Disease Care: Interrupted Time Series Study

**DOI:** 10.2196/55314

**Published:** 2024-03-22

**Authors:** Reed Taylor Sutton, Kaitlyn Delaney Chappell, David Pincock, Daniel Sadowski, Daniel C Baumgart, Karen Ivy Kroeker

**Affiliations:** 1 Division of Gastroenterology, Department of Medicine, University of Alberta, Edmonton, AB, Canada; 2 Chief Medical Information Office, Alberta Health Services, Edmonton, AB, Canada

**Keywords:** decision support system, clinical, electronic medical records, electronic health records, health record, medical record, EHR, EHRs, EMR, EMRs, decision support, CDSS, internal medicine, gastroenterology, gastrointestinal, implementation science, implementation, time series, interrupted time series analysis, inflammatory bowel disease, IBD, bowel, adherence, flare, flares, steroid, steroids, standardized care, nurse, clinical practice guidelines, chart, electronic chart, electronic medical chart

## Abstract

**Background:**

Clinical decision support systems (CDSSs) embedded in electronic medical records (EMRs), also called electronic health records, have the potential to improve the adoption of clinical guidelines. The University of Alberta Inflammatory Bowel Disease (IBD) Group developed a CDSS for patients with IBD who might be experiencing disease flare and deployed it within a clinical information system in 2 continuous time periods.

**Objective:**

This study aims to evaluate the impact of the IBD CDSS on the adherence of health care providers (ie, physicians and nurses) to institutionally agreed clinical management protocols.

**Methods:**

A 2-period interrupted time series (ITS) design, comparing adherence to a clinical flare management protocol during outpatient visits before and after the CDSS implementation, was used. Each interruption was initiated with user training and a memo with instructions for use. A group of 7 physicians, 1 nurse practitioner, and 4 nurses were invited to use the CDSS. In total, 31,726 flare encounters were extracted from the clinical information system database, and 9217 of them were manually screened for inclusion. Each data point in the ITS analysis corresponded to 1 month of individual patient encounters, with a total of 18 months of data (9 before and 9 after interruption) for each period. The study was designed in accordance with the Statement on Reporting of Evaluation Studies in Health Informatics (STARE-HI) guidelines for health informatics evaluations.

**Results:**

Following manual screening, 623 flare encounters were confirmed and designated for ITS analysis. The CDSS was activated in 198 of 623 encounters, most commonly in cases where the primary visit reason was a suspected IBD flare. In Implementation Period 1, before-and-after analysis demonstrates an increase in documentation of clinical scores from 3.5% to 24.1% (*P*<.001), with a statistically significant level change in ITS analysis (*P*=.03). In Implementation Period 2, the before-and-after analysis showed further increases in the ordering of acute disease flare lab tests (47.6% to 65.8%; *P*<.001), including the biomarker fecal calprotectin (27.9% to 37.3%; *P*=.03) and stool culture testing (54.6% to 66.9%; *P*=.005); the latter is a test used to distinguish a flare from an infectious disease. There were no significant slope or level changes in ITS analyses in Implementation Period 2. The overall provider adoption rate was moderate at approximately 25%, with greater adoption by nurse providers (used in 30.5% of flare encounters) compared to physicians (used in 6.7% of flare encounters).

**Conclusions:**

This is one of the first studies to investigate the implementation of a CDSS for IBD, designed with a leading EMR software (Epic Systems), providing initial evidence of an improvement over routine care. Several areas for future research were identified, notably the effect of CDSSs on outcomes and how to design a CDSS with greater utility for physicians. CDSSs for IBD should also be evaluated on a larger scale; this can be facilitated by regional and national centralized EMR systems.

## Introduction

Limited or delayed adoption of professional society–developed clinical care guidelines into practice is a common problem in medicine [1,2]. In 2007, researchers estimated that it took 17 years on average for only 14% of published evidence in guidelines to be translated into clinical practice [3,4]. One purported reason is that clinical guidelines by themselves are not actionable, as they largely describe what to do but not how to do it [5,6].

Clinical decision support systems (CDSSs) are tools that can be used to support provider decision-making. A CDSS uses clinical, patient, and other health information to supply providers with recommendations to assist in a variety of aspects of care, including diagnosis, treatment, and management [7,8]. Recent systematic reviews suggest that the use of CDSSs in clinical settings can improve practitioner performance in relation to adherence to best practice guidelines [7,9].

There are several demonstrated gaps in the adoption of professional society clinical care guidelines and best practices for inflammatory bowel disease (IBD). These include practices in medication management, preventative care, and bone health [10,11]. The University of Alberta IBD outpatient clinic (Edmonton, Alberta, Canada) has previously developed and implemented several clinical care pathways to consolidate best practices for IBD [10,12]. To further increase adoption, a clinical decision support (CDS) project was undertaken to integrate the pathways into the local electronic medical record (EMR). There are thousands of CDS projects built and deployed within commercial EMRs [13,14], yet there are few published evaluations of EMR-based CDSSsfor IBD [15,16]. Consequently, the objective of this pilot study was to evaluate the effectiveness and provider acceptance of an EMR-integrated CDSS in the context of IBD.

## Methods

### Ethical Considerations

This study received approval from the University of Alberta Health Research Ethics Board (Pro00083538). A waiver of informed consent was also approved as part of our study by the Health Research Ethics Board.

### Organizational Setting

The study was conducted in the Comprehensive Academic Outpatient Center at the University of Alberta Hospital, which provides care for patients with IBD in the Greater Edmonton region as well as rural and remote communities across Alberta, Canada. It also serves a small number of patients with IBD from Saskatchewan, Northwest Territories, and British Columbia.

### System Details and System in Use

The clinic’s preexisting system was an enterprise EMR based on the 2014 version of Epic EMR (Epic Systems), which was being used for outpatient medical care in Edmonton, Alberta. This system was customized and branded locally as eCLINICIAN. Medication lists, allergies, and health problems are recorded and shared between users as part of clinical documentation, order entry, and planning. The system was implemented for gastroenterology outpatient care in March 2014.

As Epic is a general-purpose EMR, it includes built-in CDS functionality. For example,this includes generic functionality, such as alerting users when duplicate orders exist. More specialty-specific CDS features are often customized at the request and guidance of end users.

Functionality can be administered through a number of tools, including those referred to by Epic as “Flowsheets” (documentation tables), “Best Practice Advisories (BPAs)” (alerts) [17], and “SmartSets” (ie, grouping of orders and clinical content) [18].

These tools, particularly BPAs and SmartSets, are clinical data and test result driven; they can be triggered by unique combinations of provider characteristics, patient demographics, test results, clinical problems, as well as current and requested medications.

### System Interruption and Intervention

**Figure 1.**
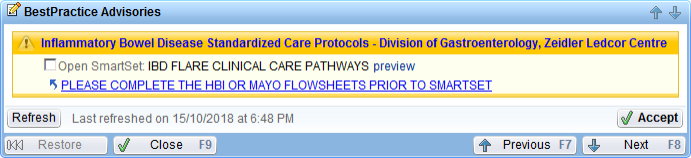
Snapshot of the inflammatory bowel disease (IBD) flare clinical decision support system, showing the initial Best Practice Advisory. Best Practice Advisories act as alerts that present targeted patient-specific guidance to users. They can be active (disruptive pop-ups) or passive (navigation workflow) and can link to actions such as placing orders, order sets, initiating a care plan, or sending a message. This alert appeared passively in the providers’ workflow navigation whenever IBD was in the patient problem list.

The system interruption and intervention uses BPA appearing in the clinician’s navigator workflow. The BPA is triggered by the existence of IBD in the patient problem list or visit diagnosis fields. The BPA (Figure 1) prompts the clinician to complete clinical symptom indices—modified Harvey Bradshaw Index (mHBI) [19] for Crohn disease or partial Mayo (pMayo) score [20] for ulcerative colitis—for the encounter. If the score is indicative of a disease flare, the BPA instructs the user to activate a corresponding SmartSet.

**Figure 2.**
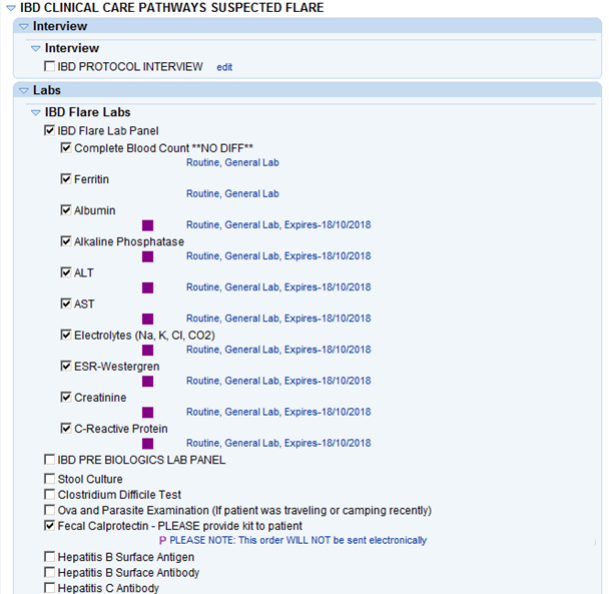
Snapshot of the inflammatory bowel disease (IBD) flare clinical decision support system, showing the SmartSet, after activation by Best Practice Advisory. Not all sections of the SmartSet are shown, including sections for medications, imaging investigations, billing, and follow-up appointment booking. ALT: alanine transaminase; AST: aspartate aminotransferase; Cl: chloride; CO2: carbon dioxide; ESR: erythrocyte sedimentation rate; K: potassium; Na: sodium; NO DIFF: no differential.

The SmartSet offers ordering and printing of appropriate lab panels, stool cultures, and other investigations, including imaging, procedures, and medication prescriptions. All recommendations were designed to be consistent with established IBD care guidelines and the flare protocol for the clinic. For example, during a flare encounter, the IBD flare lab panel and fecal calprotectin (FCP) tests are automatically selected for ordering (they can still be deselected by the provider). A snapshot of the SmartSet portion of the CDSS is shown in Figure 2.

### Study Design

The study used a pre- and postimplementation interrupted time series (ITS) design, the interruption being the enhanced CDSS used within the EMR. Each data point represented 1 month of clinical encounters. For each intervention period, there was a total of 18 data points, 9 before and 9 after the intervention. Multimedia Appendix 1 presents an elaboration on the rationale for using an ITS design.

Physicians at the participating clinic were not guaranteed to have outpatient clinics on a weekly basis due to their service rotation; therefore, it was decided to aggregate the data points by month instead of by week. This avoided the potential week-to-week variation and ensured an adequate number of individual patient encounters (IBD flares) for each data point.

The Quality Criteria for ITS Designs checklist was used in the study design and assessment of appropriateness [21], and the Statement on Reporting of Evaluation Studies in Health Informatics (STARE-HI) guidelines were used for health informatics evaluations [22,23].

### Participants

All IBD care providers at the university-based outpatient clinic were included in the study and invited to use the CDSS, including 7 IBD specialist clinicians, 1 IBD nurse practitioner, and 4 IBD specialist nurses. The term “IBD practitioner” will be used to collectively refer to IBD specialists and IBD nurse practitioners.

To be included in the data set, patients had to be under the care of the IBD providers; aged ≥18 years; and diagnosed with either Crohn disease or ulcerative colitis confirmed by imaging, pathology, or endoscopy report. They also had to be experiencing a flare of the disease during the included encounter, as defined by clinical scores (mHBI >5; pMayo >2) or noted symptoms in combination with physician judgment. Only initial encounters in a flare episode spanning multiple encounters were included.

### Study Flow

**Figure 3.**
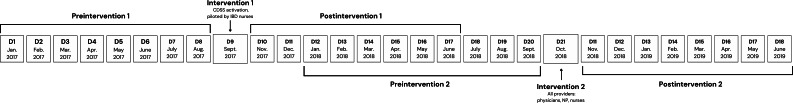
Study design diagram of the 2-period interrupted time series design. First, the clinical decision support system (CDSS) was implemented as a limited pilot with inflammatory bowel disease (IBD) nurses (intervention 1), and then, it was fully implemented across all providers (intervention 2). Each data point (abbreviated as D) corresponds to 1 month of clinical encounters by study providers. NP: nurse practitioner.

The intervention was implemented and evaluated in 2 continuous periods (Figure 3). First, a pilot version was trialed by IBD nurses (Implementation Period 1), and then, the polished version was implemented across all providers in the division (ie, clinicians, nurse practitioners, and IBD nurses) as Implementation Period 2. The pilot version was trialed beginning in September 2017 and included the following 3 SmartSets available within the BPA, corresponding to different positions along the care path of a patient with flaring IBD: suspected flare, 2 to 4 weeks into the flare, and 16 weeks’ postflare assessments. Feedback was gathered informally from providers (Multimedia Appendix 2) to inform further improvement to the CDSS.

After collecting feedback from the pilot, further changes were made to the CDSS. Aside from minor modifications to update included lab tests, the most significant change was the consolidation of the 3 separate SmartSets into 1, targeting the “suspected flare,” the first step in the care pathway. The activation of the BPA in the initial CDSS was entirely manual and relied on the provider entering a specific visit diagnosis. However, in the full version, the BPA was set to automatically trigger based on the presence of an IBD diagnosis in the patient’s problem list. This change was expected to improve the adoption and ease of use of the SmartSet for flare encounters.

The full implementation of the CDSS began on October 10, 2018. An instructional memo with paper-based workflow and educational material was sent to each provider (Multimedia Appendix 3). Over the course of 1 month, each participant was given the opportunity to ask questions about using the system and access to use the system in the sandbox environment. A demonstration of the system was also presented at weekly clinical rounds, with an opportunity to ask questions.

### Outcome Measures

Process indicators were used to measure the proportion of adherent IBD practitioner flare encounters. These indicators include completion of clinical scores (mHBI or pMayo); laboratory testing, such as standard lab panel, FCP, stool cultures, and *Clostridium difficile* toxin (only if diarrhea is present); and of vitamin D or calcium in conjunction with corticosteroid prescription, patient information given and documented, and modification of maintenance therapy. A secondary outcome was the adoption or acceptance of the CDSS measured by application rate (ratio of CDSS uses to CDSS available for activation).

### Methods for Data Acquisition and Measurement

Potential encounters in the pre- and postintervention periods were initially identified by querying the eCLINICIAN EMR database for encounters with the included IBD providers, where patients had documentation of IBD in their problem list or diagnosis field (*International Classification of Diseases* coding). A sampling method was used to exclude encounters with specific reasons for visit deemed unlikely to constitute a flare based on exploratory analysis of the data set. Examples of excluded reasons for the visit included “medication refill,” “medical insurance coverage,” and “review results” (a more detailed description of the sampling method is available in a previous publication [10]). Encounters were then screened manually for inclusion and exclusion eligibility by one of the authors (RTS) and a research assistant.

Data for primary outcome measures were also queried and extracted from the EMR database, in collaboration with the eCLINICIAN reporting team in Alberta Health Services (AHS). The various database codes and IDs as well as the final SQL queries used to extract data are included in the Multimedia Appendix 4.

### Methods for Data Analysis

Descriptive statistics were calculated to determine patient characteristics, with data presented as counts and proportions for categorical variables, mean (SD) values for normally distributed continuous variables, and median (IQR) values for nonnormally distributed continuous variables. Proportions were compared by using the Pearson *χ*^2^ test [24].

A segmented regression analysis was performed for each primary outcome variable to determine the level and slope in the preintervention period as well as the change in level and slope in the postintervention period regarding the mean percentage of adherent encounters [25]. Autocorrelation in the residuals was tested using the Durbin-Watson test.

Data analysis was performed using IBM SPSS Statistics (version 23; IBM Corp) and R 3.5.1 (RStudio Inc) [26]. A 95% CI was used in all analyses unless otherwise specified.

### Sample Size Determination

The sample size was first calculated for pre- and postimplementation cohorts based on logistic regression (Multimedia Appendix 5). With a power of 0.80 and a type I error set to 5%, the sample size required was approximately 634 for small effects and 145 for medium effects [27]. This assumes equal sample sizes (N) in the comparison groups and an initial proportion of adherence to each guideline component of approximately 70%, chosen based on a recent study by Jackson et al [11]. The sample size was calculated using G*Power 3.2.9.2 [28].

There is no standard method for determining power in time series analyses. However, a simulation-based power calculation displayed that with N=16 (8 data points in the preintervention period and 8 data points in the postintervention period), there is a 70% chance to detect an effect size of 0.5 or more, and over 90% chance to detect an effect size of 1 or more, at an alpha level of .05 [29]. It is also generally recommended in the literature to have over 100 observations per data point [25,30].

## Results

### Initial Data Set and Preprocessing

**Figure 4.**
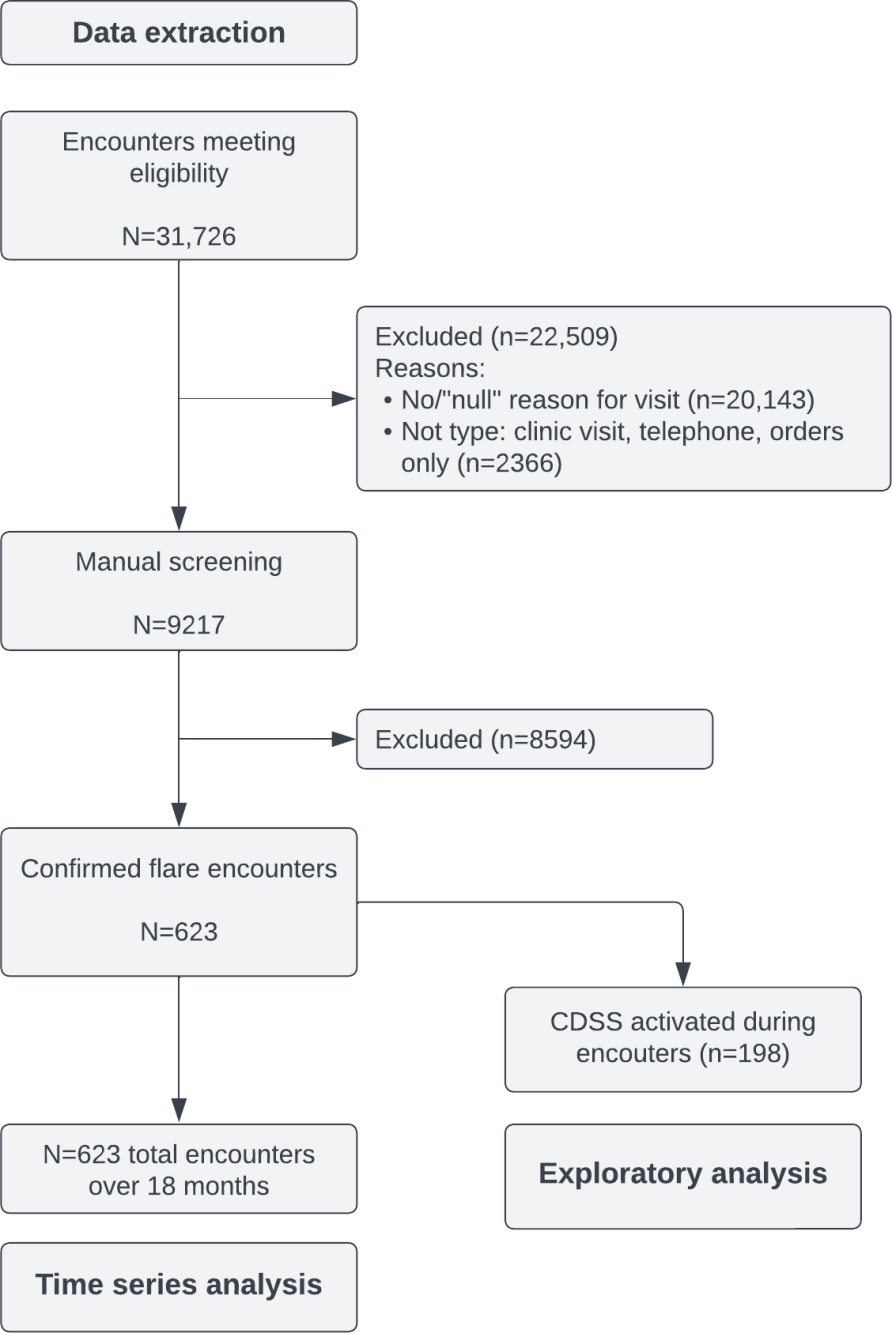
Flow data diagram for data extraction, screening, and analyses. CDSS: clinical decision support system.

Figure 4 shows the study’s flow diagram. The complete, extracted data set includes 31,726 encounters from January 1, 2017, to June 30, 2019. When considering only clinic visits (7655), orders (16,485), and telephone (5220) encounter types, the data set totals 29,360 (92.5%) encounters. There was an average of 998 encounters per month, with a minimum of 735 (December 2018) and a maximum of 1202 (May 2017) encounters. Of note, there is an overlap between both implementation periods (Figure 3), and thereby, a number of flare encounters appear in both analyses.

### Demographics of CDSS-Enabled Encounters

From September 2017 to June 2019, the CDSS was activated a total of 214 times across 214 encounters with 207 patients. Of these, 16 encounters were excluded from analysis due to, upon review, not being used appropriately for a flare or suspected flare encounter with a patient with IBD. This left 198 encounters, which are detailed in Table 1. More detailed demographics of providers using the system are included in Multimedia Appendix 6.

**Table 1.** Demographics of users and encounters invoking the inflammatory bowel disease (IBD) flare clinical decision support system.

Demographic variables	Study population (n=198)
**Provider characteristics**	
**Provider type, n (%)**	
IBD nurse	172 (86.9)
IBD practitioner	26 (13.1)
**Patient characteristics**	
**Sex, n (%)**	
Female	113 (57.1)
Male	85 (42.9)
Age (years), median (IQR)	37.5 (29-49)
**Current IBD therapy, n (%)**	
None	37 (18.7)
5-aminosalicylic acid only	53 (26.8)
Immunomodulator	18 (9.1)
Biologic monotherapy	59 (29.8)
Biologic combination therapy	31 (15.7)
**Encounter characteristics**	
**Encounter type, n (%)**	
Telephone	139 (70.2)
Orders only	32 (16.2)
Clinic visit	27 (13.6)
**First encounter diagnosis, n (%)**	
None	172 (86.9)
Crohn disease	11 (5.6)
Ulcerative colitis	10 (5.1)
Bloody diarrhea	2 (1.0)
IBD	1 (0.5)
Abdominal bloating	1 (0.5)
Ankylosing spondylitis	1 (0.5)
**Visit reason, n (%)**	
Suspected IBD flare	113 (57.1)
IBD	39 (19.7)
Disease flare-up	15 (7.6)
None	9 (4.5)
Referral	9 (4.5)
Follow-up	7 (3.5)
Diarrhea	3 (1.5)
Medication change	1 (0.5)
Medication problem	1 (0.5)

### Study Findings and Outcome Data

#### Exploratory Analysis of Adherence to Clinical Protocols

##### Symptom Documentation

Of 192 patients with clinical scores (mHBI or pMayo) that were applicable (excluding those without pouch or short bowel or those newly diagnosed), 133 (69.3%) had a clinical score completed and documented in their chart at the index dispensation. Of all 198 encounters, 196 (99.0%) had symptoms (ie, pain, number and characteristics of stool, and the presence of blood) documented in the chart by the provider.

##### Laboratory Investigations

Full flare lab panels, including complete blood count, ferritin, electrolytes, creatinine, albumin, alkaline phosphatase, alanine transaminase, aspartate transaminase, and C-reactive protein (CRP), were ordered for 109/198 (55.1%) patients exactly at the encounter. Including orders up to 1 month prior, full panels were ordered for 183/198 (92.4%) patients. However, 113/198 (57.1%) had at least a partial lab panel, including complete blood count and CRP, ordered at the encounter, and 193/198 (97.5%) had partial lab panels, including complete blood count and CRP ordered up to 1 month prior to the encounter.

FCP was ordered at the encounter for 147/198 (74.2%) patients and within 1 month of the encounter for a further 36/198 (18.2%). This leaves only 15 (7.6%) who had no evaluation of FCP at all. Furthermore, testing for *Clostridium difficile* infection was done in 164/198 (82.8%) patients and for stool cultures in 160/198 (80.8) patients. In 138 patients with liquid stool or diarrhea mentioned in the progress note, 127 (92%) had *Clostridium difficile* testing ordered and 123 (89.1%) had stool cultures ordered.

##### Provision of Steroid-Sparing Therapy and Osteoprotective Therapy

In this data set, only 12 (6.1%) patients were prescribed steroids at their encounter. Of these, 6 (50%) had maintenance IBD therapy adjusted or added. In contrast, 37 (20%) of the 185 patients who were not prescribed steroids had maintenance therapy adjusted (*P*=.02 for *χ*^2^).

Vitamin D or calcium supplementation was recommended for 8/12 (67%) patients prescribed steroids and 8/10 (80%) when excluding patients with vitamin D or calcium supplementation already documented in their medication list.

### Implementation Period 1: Pilot CDSS Version With IBD Nurses

Implementation Period 1 included data from January 2017 to June 2018 (18 months), where September 2017 and beyond were labelled as the active intervention months (postintervention). Of the total 623 confirmed flare encounters, 502 occurred during Implementation Period 1 (Figure 3). Table 2 compares outcome measures before and after the intervention using chi-square tests. Notably, there was a substantial increase in the proportion of flare encounters with completed clinical scores from 3.5% (8/228) to 24.1% (66/274) post intervention. There was also an increase in the proportion of flare encounters with FCP ordered, from 16.7% (38/228) to 27% (74/274).

**Table 2.** Before-and-after analysis of process measures from Implementation Period 1.

Parameter	Preintervention (n=228), n (%)	Postintervention (n=274), n (%)	*P* value^a^
CDSS^b^ activated	0 (0)	66 (24.1)	<.001
Clinical score completed	8 (3.5)	66 (24.1)	<.001
Flare labs ordered	124 (54.4)	132 (48.2)	.33
C-reactive protein ordered	156 (68.4)	178 (65.0)	.56
Fecal calprotectin ordered	38 (16.7)	74 (27.0)	.048
Stool cultures ordered	128 (56.1)	162 (59.1)	.63
*Clostridium difficile* test ordered	128 (56.1)	172 (62.8)	.29

a *P* value of the Pearson chi-square test comparing proportions.

b CDSS: clinical decision support system.

**Figure 5.**
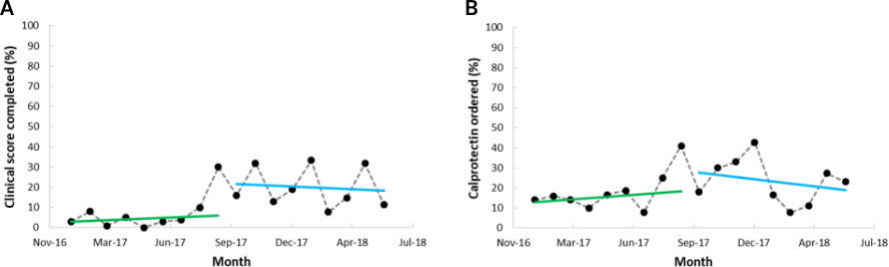
Segmented regression for Implementation Period 1 (pilot) of the inflammatory bowel disease flare clinical decision support system on rates of (A) clinical score completion and (B) calprotectin testing.

ITS analysis was done for outcomes that were significant in the before-and-after analyses (Figure 5). For clinical score completion rates, there was no slope change (estimated β −1.22, 95% CI −4.44 to 2.01*; P*=.43), but there was a level increase (estimated β 19.0, 95% CI 2.39-35.60; *P*=.03). For calprotectin testing, there was no slope change (estimated β −2.45, 95% CI −6.21 to 1.32; *P*=.19) or level change (estimated β 14.77, 95% CI −4.63 to 34.17; *P*=.13).

### Implementation Period 2: Full CDSS Implementation With All Providers

Implementation Period 2 included data from January 2018 to June 2019 (18 months), where October 2018 and beyond were postintervention months. Of the total 623 confirmed flare encounters, 492 occurred during Implementation Period 2 (Figure 3). Table 3 compares outcome measures before and after the intervention using chi-square tests. There were increases in the proportion of flare encounters with completed flare labs (109/229, 47.6% to 173/263, 65.8%), CRP ordered (147/229, 64.2% to 207/263, 78.7%), calprotectin ordered (64/229, 27.9% to 98/263, 37.3%), and stool cultures ordered (125/229, 54.6% to 176/263, 66.9%).

**Table 3.** Before-and-after analysis of process measures from Implementation Period 2.

Parameter	Preintervention (n=229), n (%)	Postintervention (n=263), n (%)	*P* value^a^
Application of SmartSets	52 (22.7)	72 (27.4)	.23
Clinical score completed	58 (25.3)	75 (28.5)	.43
Flare labs ordered	109 (47.6)	173 (65.8)	<.001
C-reactive protein ordered	147 (64.2)	207 (78.7)	<.001
Fecal calprotectin ordered	64 (27.9)	98 (37.3)	.03
Stool cultures ordered	125 (54.6)	176 (66.9)	.005
Clostridium testing ordered	136 (59.4)	177 (67.3)	.70

a *P* value of the Pearson chi-square test comparing proportions.

**Figure 6.**
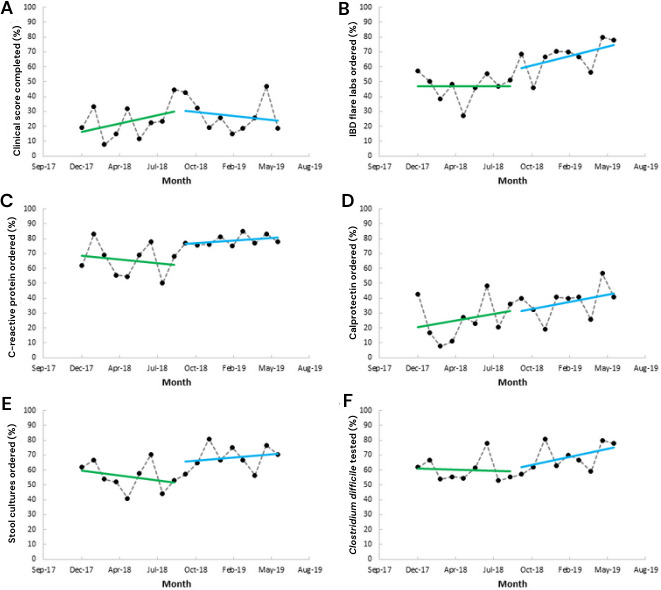
Segmented regression for Implementation Period 2 of the inflammatory bowel disease (IBD) flare clinical decision support system on rates of (A) clinical score completion, (B) flare lab testing, (C) C-reactive protein testing, (D) calprotectin testing, (E) stool culture testing, and (F) *Clostridium difficile* testing.

The ITS analysis for significant outcomes is shown in Figure 6, and accompanying β values for slope change and level change with 95% CIs are shown in Table 4. For Period 2, there were no slope or level increases that reached significance at *P*=.05, although CRP testing and stool culture testing would be significant for a level increase at *P*=.10.

**Table 4.** Parameters for segmented logistic regression analysis of the inflammatory bowel disease (IBD) clinical decision support system (CDSS) in Implementation Period 2.

Parameter		ß	95% CI	*P* value
**Application rate**				
	Preintervention slope (secular trend, per month)	0.151	−3.757 to 4.059	.94
	Change in slope (gradual effect, per month)	2.019	−3.508 to 7.546	.45
	Change in intercept (immediate effect)	−5.048	−33.86 to 23.76	.71
**Clinical scores completed and documented**				
	Preintervention slope (secular trend, per month)	1.648	−1.596 to 4.893	.29
	Change in slope (gradual effect, per month)	−2.463	−7.051 to 2.125	.27
	Change in intercept (immediate effect)	−0.992	−24.91 to 22.92	.93
**IBD flare lab tests ordered**				
	Preintervention slope (secular trend, per month)	−0.016	−2.693 to 2.662	.99
	Change in slope (gradual effect, per month)	1.929	−1.858 to 5.715	.29
	Change in intercept (immediate effect)	12.60	−7.137 to 32.34	.19
**C-reactive protein ordered**				
	Preintervention slope (secular trend, per month)	−0.742	−3.121 to 1.637	.52
	Change in slope (gradual effect, per month)	1.253	−2.111 to 4.618	.44
	Change in intercept (immediate effect)	14.89	−2.645 to 32.43	.09
**Fecal calprotectin ordered**				
	Preintervention slope (secular trend, per month)	1.298	−2.209 to 4.806	.44
	Change in slope (gradual effect, per month)	0.183	−4.778 to 5.143	.94
	Change in intercept (immediate effect)	−1.034	−26.89 to 24.82	.93
**Stool cultures ordered**				
	Preintervention slope (secular trend, per month)	−1.060	−3.650 to 1.529	.40
	Change in slope (gradual effect, per month)	1.714	−1.948 to 5.376	.33
	Change in intercept (immediate effect)	15.37	−3.715 to 34.46	.11
	Clostridium difficile ordered			
	Preintervention slope (secular trend, per month)	−0.228	−2.613 to 2.158	.84
	Change in slope (gradual effect, per month)	1.825	−1.549 to 5.198	.27
	Change in intercept (immediate effect)	3.258	−14.33 to 20.84	.70

## Discussion

### Answering the Study Question

In this study, we evaluated the effectiveness of a CDSS that aimed to standardize protocols for patients with IBD experiencing an acute disease flare. An increase in several practices was demonstrated following the CDSS implementation, including increased FCP use. Completion of clinical scores also increased during Implementation Period 1 (before-and-after analysis and ITS analysis) and remained increased throughout Implementation Period 2.

We did not reach significance in slope changes or level changes in any ITS analysis in Period 2. This could be due to the sample size, which may also account for the large variance seen in some data points. There were, however, some encouraging upward trends in flare lab testing, particularly CRP (*P*<.10 in the ITS analysis) and stool cultures.

In characterizing the adoption of this CDSS by the application rate, an interesting finding was that the CDSS was used more by IBD nurses compared to nurse practitioners. This could represent the nurses’ increased experience with the CDSS from the pilot phase and our CDSS focus on decisions related to patients experiencing a disease flare. In the University of Alberta clinic, patients are instructed to call the IBD nurse flare line if they experience changes in symptoms, and so nurses are often the first point of contact in the flare clinical pathway. This is supported by our data showing flare encounters are primarily telephone encounters. Other research has shown that flares are unlikely to coincide with scheduled clinic appointments, which aligns with the current uptake in remote monitoring and rapid access clinics [31-33].

Our observed CDSS use by specialized IBD nurses is in contrast to several other studies that have demonstrated that nurses are less likely to use CDSSs when making decisions about care they are experienced and confident in delivering, especially in the case of telephone triage decisions [34-36]. Our results could be a product of the integration of the nurses’ feedback after the pilot phase, a strategy that may have increased the utility of the CDSS for nurses. This highlights recommendations from other research that emphasize the importance of engaging all stakeholders but especially end users in the CDSS design [37,38].

### Limitations of the Study

There are several limitations to this research. Although the ITS design allows for better characterization of temporal changes compared to before-and-after analyses, it is still possible that other changes, such as clinic structure and release or dissemination of guidelines, could have led to the changes observed. However, apart from the intervention activation and the released memo and instructions for use that were disseminated, to our knowledge, there were no other educational campaigns, institutional changes, or major publications promoting the specific care guidelines investigated by the study. There were subtle changes in staff, for example, the joining of a new IBD physician and the leaving of another. However, there were no changes in IBD nurse staff, who were the primary users of the CDSS.

In contrast to the advantage of our 2-phased design regarding the opportunity for feedback from nurses, the design may have hindered our ability to demonstrate change. As we used the same group of IBD nurses in the pilot (Phase 1) and implementation (Phase 2) periods, our baseline use prior to the beginning of Phase 2 had already started. This may have accelerated the observed uptake speed of the CDSS by practitioners and could have also led to an underestimation of the changes before and after Implementation Phase 2.

Sample size is another limitation. In an ITS analysis, it is recommended to have a minimum of 16 data points and 100 observations per data point [25,29,30]. Although we met the data point requirement, the number of flares per month was consistently under 50. Future studies should aim to include more data points, which may require multisite participation. Unfortunately, at the time of this study, the EMR software was only deployed at a single site.

We only captured data from orders that were tied to the encounter. If a decision was made to not order labs for any reason (eg, they were recently completed), they would not be captured by our extraction. As a consequence, estimates of protocol adherence could be deflated.

Finally, it is important to note that for process measures that depend on manual data entry, such as clinical score completion, this research method can only determine whether a process was documented as completed but not necessarily whether it was actually completed. This may have resulted in underestimates of protocol adherence.

### Future Directions

The currently available CDSS in this study was limited in its ability to support complex multiprovider pathways and tie together multiple visits along a pathway. More advanced CDSS workflows should be investigated in future versions of the CDSS software and evaluated for effectiveness.

Triggering logic for CDSSs should also be precisely targeted. For example, a CDSS should determine whether a patient has had a test done within a certain time span, and if not, prompt the user to order it. The reverse should also be possible; if a test has been recently ordered (eg, *Clostridium difficile,* which can only be tested once every 2 weeks), the CDSS could automatically deselect or prompt the user to remove this order to save downstream resources. This was not possible with the resources available in our CDSS environment.

In extracting data for analysis, a significant challenge was identifying flare encounters based on EMR data. The problem stems from a lack of discrete data identifying patients with active diseases (clinical scores were not regularly documented as discrete data). Future research should seek to develop a case definition for disease flare through administrative provincial data sets. This could include quantitative metrics, such as CRP and FCP, that predict the likelihood of flare, but it could also include the integration of a case-finding algorithm that uses natural language processing to parse clinical notes. This strategy has been explored in several other diseases and has been shown to significantly improve case detection [39]. Some work has been done in IBD to identify phenotypic information from clinic notes using natural language processing [40].

The methodology used in this research should be expanded to investigate the effects of improved versions of CDSS for IBD on other community clinics and nonacademic practices throughout Alberta. Cluster-randomized designs or stepped-wedge designs could be explored since multiple clinics could be available for randomization.

This study did not investigate the impact on patient outcomes, which would require a longer follow-up period (ideally 2 or more years). Nonetheless, long-term patient outcomes for the CDSS are of great importance [9] and should be explored in the future.

### Conclusions

Through our study, we designed and implemented, in 2 phases, a CDSS for IBD disease flare embedded in existing EMR software and evaluated the impact of the CDSS on provider adoption of clinical guidelines and local best practices. We have shown moderate adoption and acceptance of this system by providers, particularly by IBD nurses, as measured by the system application rate. Findings from the first phase support the hypothesis that the CDSS improved the use of FCP and the documentation of clinical scores. Findings from the second phase support further improvement in ordering flare lab panels, CRP, and stool cultures, as shown in before-and-after analysis and multivariate analysis. In addition, potential improvements in workflow integration were identified through qualitative questionnaires and feedback forms; areas for future research have also been established.

## Supplementary material

10.2196/55314 Multimedia Appendix 1 The rationale for using an interrupted time series design.

10.2196/55314 Multimedia Appendix 2 Provider feedback.

10.2196/55314 Multimedia Appendix 3 Materials distributed to providers.

10.2196/55314 Multimedia Appendix 4 eCLINICIAN query information.

10.2196/55314 Multimedia Appendix 5 Sample size calculation.

10.2196/55314 Multimedia Appendix 6 Demographics of users (inflammatory bowel disease nurses and practitioners).

## Abbreviations

AHS: Alberta Health Services
BPA: Best Practice Advisory
CDS: clinical decision support
CDSS: clinical decision support system
CRP: C-reactive protein
EMR: electronic medical record
FCP: fecal calprotectin
IBD: inflammatory bowel disease
ITS: interrupted time series
mHBI: modified Harvey Bradshaw Index
pMayo: partial Mayo
STARE-HI: Statement on Reporting of Evaluation Studies in Health Informatics
