# Supplementary material for: The Effect of an Electronic Medical Record–Based Clinical Decision Support System on Adherence to Clinical Protocols in Inflammatory Bowel Disease Care: Interrupted Time Series Study
Source: JMIR Med Inform. 2024 Mar 22;12:e55314. doi: 10.2196/55314 (PMC11004614; doi:10.2196/55314)
Supplement: Multimedia Appendix 1 [file medinform-v12-e55314-s001.docx]

Appendix sourced from:

Sutton RT. Adherence to clinical care protocols for inflammatory bowel disease and evaluation of a clinical decision support system to improve adherence. Published online December 2019. <https://doi.org/10.7939/r3-exjr-bc17>

### Interrupted Time-series Design

The ITS is a quasi-experimental design tracks outcome measures at multiple time points (‘data points’) before and after the intervention (‘interruption’). A minimum of three time points before and three after must occur to be considered a true ITS by EPOC [22]. The ITS is useful for interventions that produce rapid, sustained impact on outcomes.

### Justification of Use in this Study

ITS provides an advantage over the BA design since secular trends can be accounted for, which might otherwise confound the analysis and weaken the ability to make conclusions [23].

For this evaluation, we considered an experimental cluster randomized design (C-RCT), as well as variations such as the cluster randomized crossover, but did not select them for several reasons, including:

1. The intervention is currently limited by the availability of the EpicCare EMR to only one gastroenterology clinic, meaning the clusters would be physician practices at a single site. While clustering by physicians is logical (since the intervention is ultimately targeting their behavior), cluster randomized studies already struggle to ensure balance across participant characteristics. The chance of high variability between clusters with such a small sample (<8) is high.
2. Balance between clusters in terms of individual N is also unlikely, as a nurse practitioner sees most flaring patients under the care of all the physicians.
3. Furthermore, the setup of the clinic may predispose a C-RCT to contamination. Often during a patient’s encounter in clinic, they will see both a physician and IBD specialist nurse. It would be infeasible to ensure that physicians randomized to one treatment were not seeing patients then seen by an IBD nurse randomized to the other treatment.
4. Finally, sample size simulations using R (clusterPower package^16^) determined that there would not be enough IBD flare encounters to reach adequate power for the C-RCT with 7-8 clusters.

### Limitations of the ITS Design

One limitation of the ITS design is the need for a significant number of repeated data points [25]. For this reason, the design lends itself to routinely measured data. In the case of our study, this data is already recorded in the medical record database automatically.

Two other limitations of this design are the lack of a true experimental control group, and the inability to draw inferences regarding individual level outcomes [25]. The former has already been discussed, and the latter is acceptable, since the goal of the assessment is to validate the intervention for use at other clinics and practices throughout the province.

There are several biases that can occur in health technology interventions and assessments. One is the ‘Hawthorne effect’, which is the tendency for humans to improve their performance when they know their behavior is being studied [26]. This is a possibility we could not rule out in this study. However, a Waiver of Consent was requested and approved by the University of Alberta Health Research Ethics Board (HREB), which should minimize any potential impact.

Another confounder and data collection bias is the ‘checklist effect’, where improvement can occur due to better structured data collection (which often occurs when computerized systems are implemented) [26]. For our study, we are not particularly concerned with separating these issues. We consider potentially improved and better structured documentation to be both part of the intervention as well as an acceptable outcome.

Finally, the data completeness effect may occur since the intervention itself collects data on the measures of interest. However; the EMR ultimately logs the same data as collected by the intervention, and so, where possible, data will be extracted from the EMR system log and not the log produced by the CDSS.

1. Talmon J, Ammenwerth E, Brender J, de Keizer N, Nykänen P, Rigby M. STARE-HI-Statement on reporting of evaluation studies in Health Informatics. Int J Med Inf; 2009;78(1):1-9. doi:10.1016/j.ijmedinf.2008.09.002
2. Brender J, Talmon J, de Keizer N, Nykänen P, Rigby M, Ammenwerth E. STARE-HI – Statement on reporting of evaluation studies in Health Informatics. Appl Clin Inform; 2013;4(3):331-358. doi:10.4338/ACI-2013-04-RA-0024
3. Pearson K. X. On the criterion that a given system of deviations from the probable in the case of a correlated system of variables is such that it can be reasonably supposed to have arisen from random sampling. Philos Mag Ser 5; 1900;50(302):157-175. doi:10.1080/14786440009463897
4. Wagner AK, Soumerai SB, Zhang F, Ross-Degnan D. Segmented regression analysis of interrupted time series studies in medication use research. J Clin Pharm Ther; 2002;27(4):299-309. doi:10.1046/j.1365-2710.2002.00430.x
5. Muggeo VMR. segmented: An R package to fit regression models with broken-line relationships; 2008;8:7.
