# Supplementary material for: The Effect of an Electronic Medical Record–Based Clinical Decision Support System on Adherence to Clinical Protocols in Inflammatory Bowel Disease Care: Interrupted Time Series Study
Source: JMIR Med Inform. 2024 Mar 22;12:e55314. doi: 10.2196/55314 (PMC11004614; doi:10.2196/55314)
Supplement: Multimedia Appendix 2 [file medinform-v12-e55314-s002.docx]

| **SmartSet Affected** | **Comment / Issue / Problem** |
| --- | --- |
| **SUSPECTED**  **MID-Flare**  **POST-Flare** | Completely remove ESR – no longer orderable |
| **SUSPECTED**  **MID-Flare**  **POST-Flare** | Automatically have stool culture, c.diff , FCP checked?  For SUSPECTED only |
| **SUSPECTED**  **MID-Flare**  **POST-Flare** | IBD Flare panel – doesn’t need to be auto checked at 2-4 weeks, not necessarily done – case by case. |
| **SUSPECTED**  **MID-Flare**  **POST-Flare** | Prebiologic panel – not auto checked – not always done – people may accidentally order |
| **SUSPECTED**  **MID-Flare**  **POST-Flare** | TB Skin Test – Does not print – have link to website form – nurses have to leave encounter to access these on the EPIC front page links |
| **SUSPECTED**  **MID-Flare**  **POST-Flare** | POST-FLARE has consultations for biologic therapy + others – this should be on SUSPECTED and MID as well. |
| **SUSPECTED**  **MID-Flare**  **POST-Flare** | CONSULTATIONS dropdown – IBD clinic link – instead of going to resources – have a link also going directly to Biologic page with all Biologic forms |
| **SUSPECTED**  **MID-Flare**  **POST-Flare** | Referral to Gastro Small Bowel– link to from IBD Clinic website |
| **SUSPECTED**  **MID-Flare**  **POST-Flare** | Link biologics to biologics summary page  Link CS to “online initiation of CS CCP” |
| **SUSPECTED**  **MID-Flare**  **POST-Flare** | DIARRHEA?   - Stool culture - C. difficile test   RECENT TRAVEL OR CAMPING?   - Ova and Parasite |
| **SUSPECTED**  **MID-Flare**  **POST-Flare** | Merge to a single BPA as the following:   “Click to complete HBI or PMAYO Flowsheet”  i) SUSPECTED FLARE  ii) MID FLARE ASSESSMENT  iii) POST-FLARE FOLLOW UP  If not possible, remove mid-flare and post-flare based on provider feedback . |

**CDSS Pilot - Feedback from IBD Staff**
