# Supplementary material for: The Effect of an Electronic Medical Record–Based Clinical Decision Support System on Adherence to Clinical Protocols in Inflammatory Bowel Disease Care: Interrupted Time Series Study
Source: JMIR Med Inform. 2024 Mar 22;12:e55314. doi: 10.2196/55314 (PMC11004614; doi:10.2196/55314)

## Materials Distributed to Providers

IBD FLARE Clinical Decision Support Tools

The following workflow describes the process for using the IBD Flare CDS Tool in ***e*CLINICIAN** as completed by the GI Physician or Nurse. These tools are designed in accordance with current guidelines and best practices for IBD patients with active disease.

1. In an **Encounter** with *any* IBD patient, a **BestPractice Advisory** will trigger and display as shown:


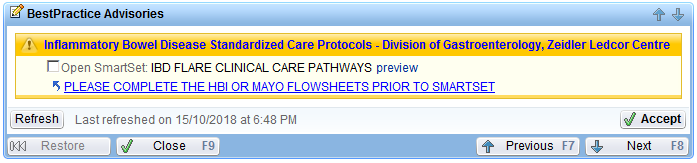


1. The BPA will prompt you to first complete the mHBI or pMAYO score flowsheet. Click on the blue link as shown:


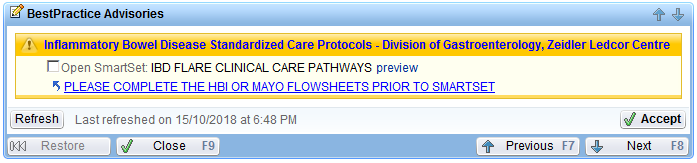


1. The hyperlink takes you directly to the **Flowsheet** activity. Fill out the appropriate clinical scale.

*(These should have been added to your default flowsheet template already, and display automatically).*


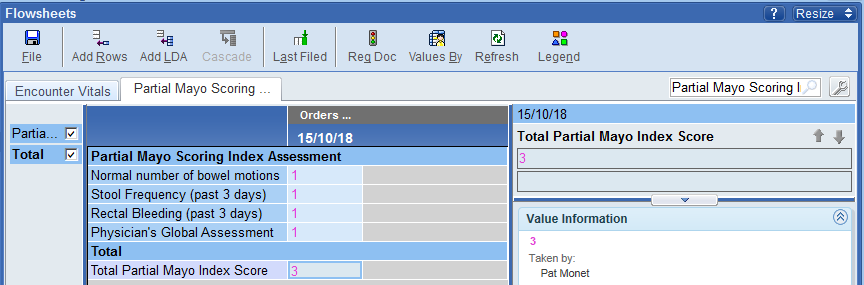


1. If your patient is suspected to be an IBD flare, return back to the Best Practice Advisory to complete the protocol, via the **Visit Navigator** activity tab.
2. Open the suggested SmartSet under the SmartSet section, by checking the box and then **Accept**.


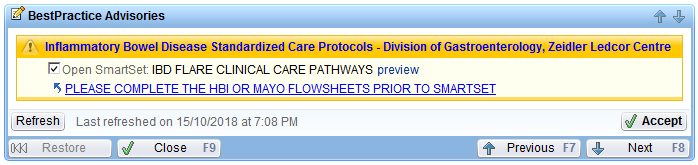


1. The IBD CLINICAL CARE PATHWAYS **SmartSet** opens. Complete the appropriate **Sections** below:

**Section I: Labs**

*Selected by default:* IBD Flare Lab Panel, Stool Culture, Clostridium Difficile, Fecal calprotectin

**Section II: Imaging, Section III: Procedures, Section IV: Medications, Section V: Consultation (Referrals), Section VI: Billing**

*Available as needed.*

**Section VII: Follow-up.** Select an option and click **Edit**


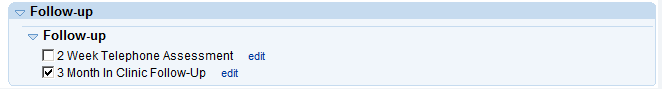


This section is for scheduling follow up encounters:

1. Mid-flare Assessment – Select to request a follow-up call re. the patient’s status (following a treatment change, for example).

Use the IBD Nurse Pool, available as **“UAH ZLC GASTRO IBD NURSES”**

1. 3 Month in Clinic Follow-up – Select to request follow-up appointment to be scheduled by your admin.
2. To send an In Basket message (CC’d Chart Message) to an individual or Pool, indicate this in the field below. You can “Personalize” a **quick list** by using the ‘Add My List’ functionality.


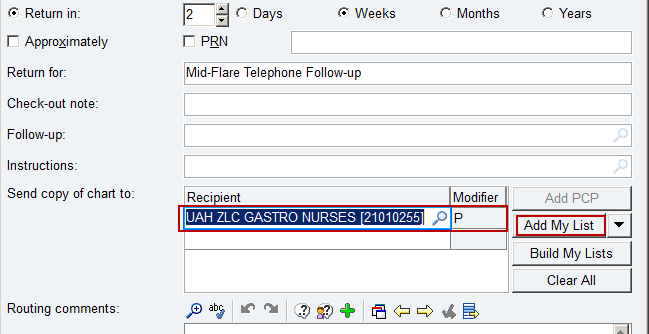


1. Click Accept to return to the **SmartSet**
2. Sign and close **SmartSet**

- Any lab tests ordered will print requisition(s) in the exam room (other than FCP, etc).
- Any medications ordered will print prescription(s) in the exam room.
- Billing will be sent automatically to the billing application.
- The Endoscopy procedures will fall on the Schedulable Orders Report.
- Follow Up – In Basket Message will be sent to the recipient(s) you have identified

| **pMAYO Total Only:** | **pMAYO Full Sheet:** | **mHBI Total Only:** | **mHBI Full Sheet:** |
| --- | --- | --- | --- |
| **.pmayototal** | **.pmayofull** | **.hbitotal** | **.hbifull** |

PULLING GI SCALES INTO TEMPLATES

The GI Scales Flowsheets for mHBI and pMAYO, when filled out, collect discrete data that can be pulled in and used in your notes, smartphrases, and templates. The following outlines an example of how to do this.

1. Go to **SmartPhrase Manager:**


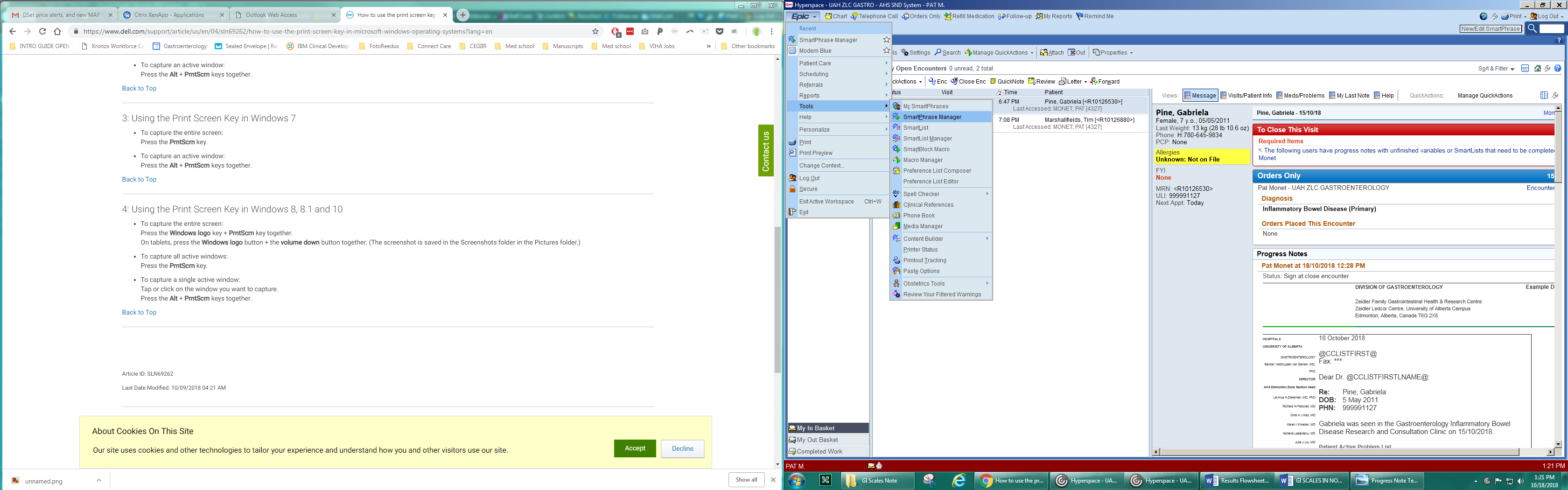


1. Your name should already be auto-filled. From there, select **‘Go’** and proceed to find your current letter/note that you want to modify:


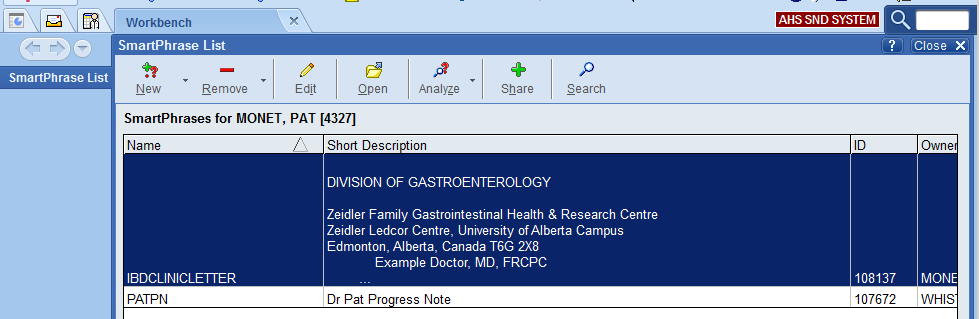


1. Double click and enter the note for editing. From there, you can add in the appropriate **SmartLinks**, which will pull in the flowsheet data from the respective score (mHBI or pMAYO).

| SmartLinks: | **pMAYO Total Only:** | **pMAYO Full Sheet:** | **mHBI Total Only:** | **mHBI Full Sheet:** |
| --- | --- | --- | --- | --- |
|  | **@FLOW(1991)@** | **@REVFS(1411:2)@** | **@FLOW(2117)@** | **@REVFS(1410:2)@** |

1. Side by side note editing and the result:

**Editor**

**The Output:**

*
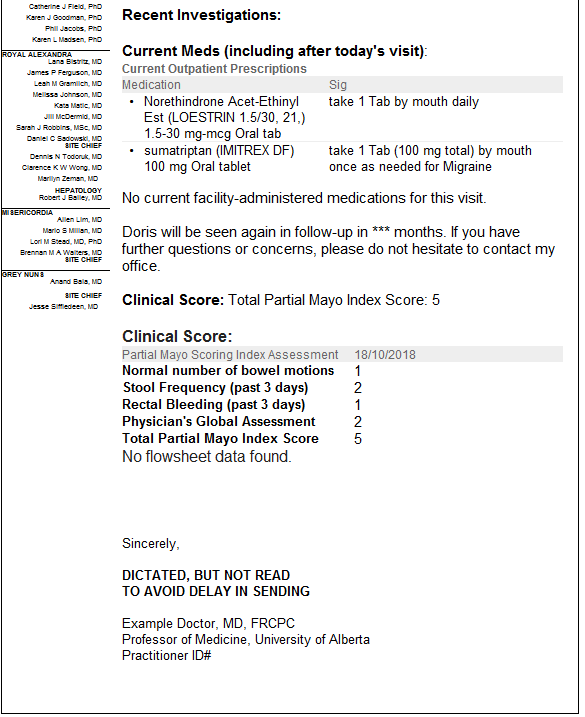

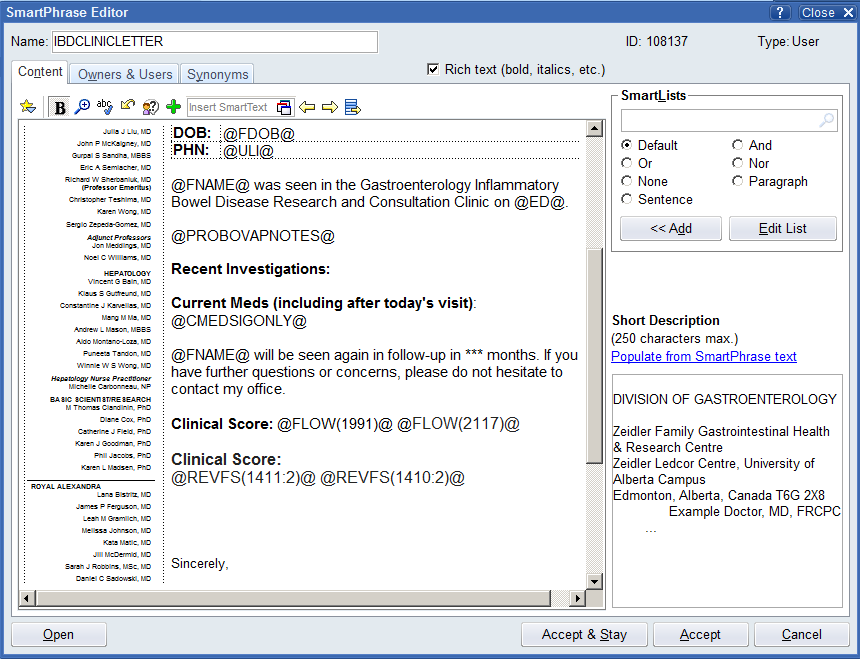
*

***Option 2: Show the full flowsheet***

***Option 1: Only show the total score***

PULLING GI SCALES REAL TIME

**We have created and shared 4 *SmartPhrases* to pull in the pMAYO and mHBI scores into *any* note in real time (ie. you do not have to pre-program them into your templates). These can be done using the ‘dotphrases’ as shown below**

| **pMAYO Total Only:** | **pMAYO Full Sheet:** | **mHBI Total Only:** | **mHBI Full Sheet:** |
| --- | --- | --- | --- |
| **.pmayototal** | **.pmayofull** | **.hbitotal** | **.hbifull** |

**Personalize! Set the flowsheet to always appear**

1. While in the **Flowsheets** activity, click on the **wrench** icon
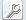
 on the very far right.


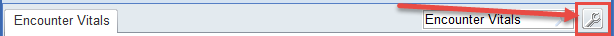


1. Check the checkbox **Override Template Order**.
2. Click in the first empty row in the Template Column and select the **magnifying glass**.
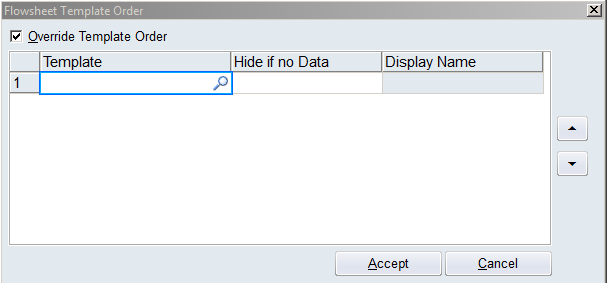

3. Under Preference List (F5), select the flowsheet **Harvey Bradshaw Index**. You may need to search for it.
4. Accept

**For all future encounters** with any patient, a tab with the **Harvey Bradshaw Index** flowsheet will now be

available.


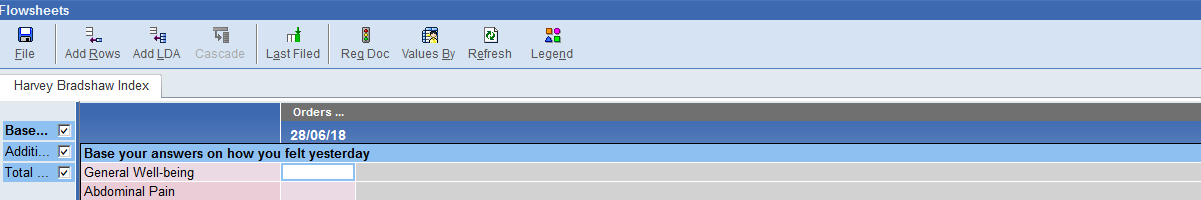


1. You will need to repeat the steps for **Partial Mayo Scoring Index Assessment.**
2. Because we overrode the template, you will also need to add **Encounter Vitals** if you require it (note: Encounter Vitals will be located under **Facility Pref List (F6)**, not **Preference List (F5):**


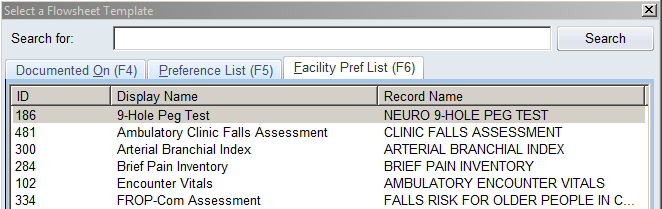

Supplement: Multimedia Appendix 3 [file medinform-v12-e55314-s003.docx]
