# Supplementary material for: The Effect of an Electronic Medical Record–Based Clinical Decision Support System on Adherence to Clinical Protocols in Inflammatory Bowel Disease Care: Interrupted Time Series Study
Source: JMIR Med Inform. 2024 Mar 22;12:e55314. doi: 10.2196/55314 (PMC11004614; doi:10.2196/55314)
Supplement: Multimedia Appendix 4 [file medinform-v12-e55314-s004.docx]

## Appendix: eCLINICIAN Query Information

### Laboratory Testing Identification

|  | |
| --- | --- |
| **ID** | **Test Name** |
| LAB 2365 | TB Skin Test- Does Not Print |
| LAB472 | Hepatitis B Surface Antibody |
| LAB2271 | Thiopurine Metabolites (6-TG and 6-MMP) |
| LAB2308 | Infliximab Antibody Level |
| LAB258 | Ova and Parasite Examination (If patient was traveling or camping recently) |
| LAB1510 | Hepatitis A Immunity (IgG & IgM) |
| LAB1296 | Hepatitis C Antibody |
| LAB1304 | HIV Antibody |
| LAB2310 | Anti-Adalimumab Antibodies |
| LAB149 | C-Reactive Protein |
| LAB223 | Stool Culture |
| LAB68 | Ferritin |
| LAB294 | Complete Blood Count **NO DIFF** |
| LAB2307 | Infliximab Trough |
| LAB66 | Creatinine |
| LAB112 | Alkaline Phosphatase |
| LAB471 | Hepatitis B Surface Antigen |
| LAB132 | ALT |
| LAB322 | ESR-Westergren |
| LAB2309 | Quantitative Analysis of Adalimumab |
| LAB2366 | Fecal Calprotectin - PLEASE provide kit to patient |
| LAB16 | Electrolytes (Na, K, Cl, CO2) |
| LAB45 | Albumin |
| LAB131 | AST |
| LAB253 | Clostridium Difficile Test (Testing will only be performed if stool is not formed) |
| LAB258 | Ova and Parasite Examination (If the patient was traveling or camping recently) |

### SQL Query for Data Extraction
