# Supplementary material for: The Effect of an Electronic Medical Record–Based Clinical Decision Support System on Adherence to Clinical Protocols in Inflammatory Bowel Disease Care: Interrupted Time Series Study
Source: JMIR Med Inform. 2024 Mar 22;12:e55314. doi: 10.2196/55314 (PMC11004614; doi:10.2196/55314)
Supplement: Multimedia Appendix 5 [file medinform-v12-e55314-s005.docx]

The sampling unit was the IBD clinic, University of Alberta, Edmonton, Canada. Sample size was first calculated for pre- and post-implementation cohorts based on logistic regression. Due to the multi-component nature of measurement of physician guideline adherence, it is expected that there will be various effect sizes, with small (OR=1.68) or medium (OR=3.47) being most common. With power equal to 0.80, Type I error set to 5%, the sample size required is approximately 634 for small effects, and 145 for medium 56 effects. This assumes equal N in the comparison groups, and an initial proportion of adherence to each guideline component of approximately 70%, which was chosen based on a recent study by Jackson et al. This sample size calculation was determined using G*Power. 3.2.9.2.

Power calculation is difficult to approximate accurately in time series, and currently there are no standardize methods or established best practices. It is generally accepted that the more data points and observations within each data point is better. A power calculation from a simulation study does offer some guidance here. The simulation-based power calculation displayed that with N of 16 (8 data points in the pre-intervention period and 8 data points in the post-intervention period), there is 70% chance to detect an effect size of 0.5 or more, and over 90% chance to detect an effect size of 1 or more, at alpha=0.05. For example, a level change of 0.20 from a baseline level of 0.5 is approximately 80% powered.

It is also generally recommended in the literature to have over 100 observations per data point. The power is also inversely proportional of the degree of auto correlation.
