# Supplementary material for: The Effect of an Electronic Medical Record–Based Clinical Decision Support System on Adherence to Clinical Protocols in Inflammatory Bowel Disease Care: Interrupted Time Series Study
Source: JMIR Med Inform. 2024 Mar 22;12:e55314. doi: 10.2196/55314 (PMC11004614; doi:10.2196/55314)
Supplement: Multimedia Appendix 6 [file medinform-v12-e55314-s006.docx]

| **Table S1.** Demographics of users (IBD nurses and practitioners). | |
| --- | --- |
| **Demographic variable** | Study population |
|  | n (% of N (11)) |
| Provider Type  IBD nurse  IBD practitioner | 4 (36.4)  7 (63.6) |
| Sex  Female  Male | 7 (63.6)  4 (36.4) |
| Length of time as healthcare provider, yrs  1-2  5-6  8-10  >10 | 1 (9.1)  1 (9.1)  2 (18.2)  7 (63.6) |
| Length of time using eCLINICIAN, yrs  0-1  1-2  3-4  >4 | 1 (9.1)  1 (9.1)  5 (45.5)  4 (36.4) |
| Length of time using any EMR, yrs  3-4  5-6  7-8  >8 | 2 (18.2)  2 (18.2)  2 (18.2)  5 (45.5) |
